# Household expenditure on non-Covid hospitalisation care during the Covid-19 pandemic and the role of financial protection policies in India

**DOI:** 10.1186/s13690-022-00857-8

**Published:** 2022-04-02

**Authors:** Samir Garg, Kirtti Kumar Bebarta, Narayan Tripathi

**Affiliations:** State Health Resource Centre, Chhattisgarh, Raipur India

**Keywords:** Access, Spending, Expenditure, Hospital, Covid, Pandemic, Private, Public, Purchasing

## Abstract

**Background:**

Despite global guidance for maintaining essential non-Covid health services during the pandemic, there is a concern that existing services faced a major disruption. The access as well as affordability of healthcare could have suffered during the pandemic, especially in developing countries including India. There are no population based studies available in India on changes in access and financial risk for non-Covid hospitalisation during the pandemic. India has a policy of Publicly Funded Health Insurance (PFHI) to ensure access and financial protection for hospital care but no information is available on its performance during the pandemic. The current study was aimed to find out the change in access and financial protection for non-Covid hospitalisations during the Covid-19 pandemic and to examine the performance of PFHI in this context.

**Methods:**

Panel data was analyzed, from two rounds of annual household surveys conducted in Chhattisgarh state for year 2019 and 2020. The survey followed a two-stage population based sample of around 3000 households, representative for the state. Two kinds of measures of catastrophic health expenditure were used – based on annual household consumption expenditure and on non-food consumption expenditure. Multivariate analysis was carried out to find determinants of utilisation and spending. In addition, Propensity Score Matching method was applied to find effect of PFHI schemes.

**Results:**

Utilisation of hospital care per 1000 population reduced from 58.2 in 2019 to 36.6 during the pandemic i.e. in 2020. The share of public hospitals in utilisation increased from 60.1% in 2019 to 67.0% in 2020. Incidence of catastrophic expenditure was significantly greater during the pandemic. The median Out of Pocket Expenditure (OOPE) in private hospitals doubled from 2019 to 2020. The size of OOPE and occurrence of catastrophic expenditure were significantly associated with utilisation in private hospitals. Enrolment under PFHI schemes including the Ayushman Bharat-Pradhan Mantri Jan Arogaya Yojana (PMJAY) was not effective in reducing OOPE or catastrophic expenditure.

**Conclusion:**

While the utilisation of hospital care dropped during the pandemic, the private hospitals became further unaffordable. The government policy for financial protection through health insurance remained ineffective during the pandemic.

**Supplementary Information:**

The online version contains supplementary material available at 10.1186/s13690-022-00857-8.

## Background

The Covid-19 pandemic has caused significant disruption in multiple sectors including healthcare [1]. Gaps in availability of healthcare for the Covid-19 infections in developing countries have received a lot of attention from researchers [2]. Some have also raised the issue of decline in non-Covid care during the pandemic [3]. Most of the existing healthcare services faced disruptions during the pandemic. The World Health Organisation (WHO) had also recognised this problem and appealed to countries to ensure the continuity of essential healthcare services during the pandemic [4]. Despite the global guidance for maintaining essential non-Covid health services, the number of individuals receiving healthcare has declined for wide ranging healthcare needs, including maternal and neonatal care, child illnesses, communicable diseases, non-communicable diseases (NCDs) and, injuries and emergencies requiring critical care [5].

A systematic review of utilisation of healthcare across 20 countries reported an average decline of one-third in utilisation. According to this review, the decline was sharper for less severe illnesses. The median decline in hospital admissions was of 28% [5]. Hospital based studies from Italy, South Korea, Croatia and USA have reported a decline in hospitalisations during the pandemic including for the non-elective conditions [6–10]. In response to the Covid-19 pandemic, the governments in Low and Medium Income Countries (LMICs) including India imposed stringent lockdowns [1]. Also with their poorly-funded and over-burdened health-systems, LMICs were more likely to experience big gaps in access to healthcare during the pandemic [11, 12]. Hospital based studies in India have reported fewer admissions including for emergency and maternal care [13–15]. The above studies covered a single or a small number of hospitals and lacked representativeness. An online survey of diabetes patients has reported worsening access and continuity of healthcare [16].

An important concern is that the non-Covid healthcare became unaffordable for the people due to the pandemic. Two studies of LMICs covered stakeholder views on non-Covid services during the pandemic [17, 18]. They have reported the stakeholders’ concern that services became difficult to access and afford. However the above studies do not provide any quantitative information on increase in cost. A survey on essential services conducted in 112 countries by the WHO reported significant disruptions of services including inpatient care in many countries [19]. The above survey did not provide any information on change in affordability of services. Populations in LMICs including India could be the worst affected due to their high levels of poverty and existing dependence on Out of Pocket Expenditure (OOPE) for financing healthcare [2, 17, 18]. There is very little information available on OOPE and Catastrophic Health Expenditure (CHE) for non-Covid care in India during the pandemic. There are no population based studies available in India on changes in utilisation and household spending for non-Covid healthcare care during the Covid-19 pandemic. Indian government has implemented a policy to ensure access and financial protection for hospital care in India but no information is available on its effectiveness during the pandemic [20].

In order to protect the households from OOPE in hospitals, the national and state governments in India have implemented Publicly Funded Health Insurance (PFHI) for more than a decade now [21]. These demand side funding mechanisms promise free hospital care to the households enrolled under the scheme. The government enrolls the households and they do not have to pay any premiums. PFHI is implemented by the government directly or by contracting an insurance intermediary. It involves empanelment of private and public hospitals [21]. The government defines a set of ailments covered under the scheme and the rates at which hospitals get reimbursed for providing treatment. All drugs, tests and procedures and stay and food of patient are covered under the benefit package. The empanelled hospitals enter into a contract with the government that prohibits them from taking any charges from the patients enrolled under PFHI [21].

India has a mixed health system with a sizeable presence of private sector healthcare providers. Private hospitals accounted for 30% of the inpatient volume for maternal care and 55% for other hospitalisations [22]. A large proportion of private hospitals are located in urban areas. The public hospitals are expected to provide most of the healthcare free of cost or at nominal user charges. Maternal care in public hospitals is completely exempt from any user fees. There is a free essential drug policy in most Indian states which mandates that patients using public facilities receive the prescribed drugs free of cost [23].

During the pandemic, the government diverted many of its hospitals for treating patients for the Covid-19 infection. In addition, some new or make-shift public facilities were built to focus exclusively on the Covid-19 patients. The government policies on maintaining essential healthcare services during the pandemic mandated the continuation of primary care services like immunisation and ante-natal care. Hospitals were expected to continue admissions for child birth and neonatal care. The elective surgeries however were discontinued. It included services for cataract, family planning and orthopedic care. There were no restrictions on private hospitals regarding non-Covid hospitalisations.

The current study was aimed to find out the change in access and financial protection for non-Covid hospitalisations during the Covid-19 pandemic and to examine the performance of PFHI in this context.

## Materials and methods

### Study setting

Chhattisgarh is one of the poorest states in India. It had a population of around 30 million in 2020, three-fourth of which resided in rural areas. Chhattisgarh recorded around 310,000 cases of Covid-19 during the year 2020 [24]. This translated into around 10,400 Covid-19 cases per million population in 2020. This was greater than the national average of 7300 per million for India and similar to the global average of 10,800 Covid-19 cases per million in 2020 [25]. Home isolation was allowed for mild or asymptomatic cases [26]. Public hospitals were mandated to provide free care for Covid-19. Elective surgeries were suspended in public hospitals. Regarding hospitalisations of non-Covid cases by private hospitals, there were no changes in policies from the pre-pandemic period. From August 2020 onwards, private hospitals were allowed to manage inpatients with Covid-19 infections [27].

Chhattisgarh’s population had nearly universal coverage under PFHI schemes that include the Ayushman-Bharat Pradhan Mantri Jan Arogaya Yojana (PMJAY), the national flagship scheme [23, 28]. Both private and public hospitals were empanelled to provide services. The schemes included ‘cash-less’ i.e. free hospital-care for 1370 medical conditions and procedures covering all medical expenses [20, 23].

### Study design and sampling

The study used panel data from annual household surveys. Annual household surveys are conducted by State Health Resource Centre, a technical agency working for the Department of Health in government of Chhattisgarh. These surveys collect data on morbidity, hospital utilisation over last one year and OOPE incurred. The first such round was carried out in November–December 2019. The survey was repeated in November–December 2020 with the same sample households. Quality assurance measures were implemented during both rounds of survey.

The survey followed a two-stage population based sample of 3000 households, representative for the state. The survey covered 15,470 individuals in 2019 and 14,926 in 2020. For a detectable difference of 5% at 95% confidence, a requirement of around 500 hospitalisations was calculated. The actual number of hospitalisation episodes that got covered in the survey was 924 and 549 for the year 2019 and 2020 respectively. The sample size available was adequate to detect a difference of 5%. In the 2020 round, there were 16 cases of Covid-19 and they were excluded from the analysis considering the focus of this study on non-Covid hospitalisations.

The change in access to hospital care was assessed in terms of utilisation of hospital care. Out of Pocket Expenditure (OOPE) was calculated for each episode by adding medical expenses and expenses on transportation and deducting any cash-reimbursements received by the patient. OOPE amount for 2020 was adjusted at 2019 prices for valid comparison. For the above adjustment, price deflators for rural (agricultural labour) and urban areas (industrial workers) were used [29].

The survey collected data on usual monthly consumption expenditure on food and non-food purposes. The two were added to calculate the total usual monthly consumption expenditure. Financial Protection was measured in terms of Catastrophic Health Expenditure (CHE) as proposed by Wagstaff and Doorslaer [30]. This study used two ways of measuring CHE:

- CHE as a proportion of annual non-food consumption expenditure: The usual monthly non-food consumption expenditure was multiplied by twelve to calculate the usual annual non-food consumption expenditure. A threshold of 40% of concerned household’s Annual Non-food Consumption Expenditure were taken for CHE and named CHE40. This is a commonly used measure of calculating CHE [30].
- CHE as a proportion of annual consumption expenditure: The total usual monthly consumption expenditure was multiplied by twelve to calculate the Usual Annual Consumption Expenditure. Thresholds of 10% and 25% of concerned household’s Annual Consumption Expenditure were taken for CHE and named CHE10 and CHE25 respectively. Recent studies in India have used the same procedure for calculating Annual Household Consumption Expenditure [23, 42].

The survey data was analysed using STATA V.15. Multivariate analysis was carried out to find determinants of utilisation, OOPE and CHE. Year of survey was included as a variable to represent the periods of pandemic (year 2020) and pre-pandemic (year 2019). The list of variables in the study is given in Supplementary Information File, S1.

Multi-variate logistic analysis was used to find determinants of utilisation of hospital care. Ordinary Least Squares (OLS) was applied for OOPE and Probit model was used for CHE. For robustness, Propensity Score Matching [PSM] was also used for evaluating effect of insurance on OOPE and CHE, as done by some recent studies in India [23, 42].

Significance was taken at 95% (p < 0.05).

The surveys were approved by the Institutional Ethics Committee of State Health Resource Centre, Chhattisgarh. Due precautions were implemented during the data collection in 2020.

### Results

The socio-demographic characteristics of the sample are given in Supplementary Information File, S2. The mean household expenditure and the mean non-food household expenditure declined from 2019 to 2020. The proportion of the formally employed declined while that of self-employed increased from 2019 to 2020.

### Utilisation of hospital care

In 2019, 58.2 hospitalisations had taken place per 1000 population and it declined to 36.6 per 1000 in 2020. As compared to 2019, the utilisation of inpatient care declined by 37% in 2020 (Table 1). The decline was less pronounced in case of maternal care as compared to the average. The decline was sharper in case of injuries and communicable diseases (Table 1).

**Table 1:** Utilisation of Hospital Care per 1000 population with 95% CI

**Disease Category**	**Annual No. of Hospitalisation episodes per 1000 population**	
	**2019**	**2020**
Communicable Diseases	17.5 [15.5–19.7]	9.3 [7.9–11.1]
Non Communicable Diseases	16.4 [14.6–18.6]	11.3 [9.7–13.1]
Maternal Care	15.6 [13.7–17.6]	12.6 [10.9–14.5]
Injuries	4.7 [3.8–5.6]	2.1 [1.5–3.1]
Others	4.0 [3.0–5.1]	1.2 [0.8–1.9]
**Overall**	**58.2 [54.7–62.1]**	**36.6 [33.8–39.8]**

Logistic regression to find determinants of utilisation showed that it was significantly likely to be lower during the pandemic (year 2020), as compared to 2019 (Supplementary Information File S3). Utilisation was likely to be lower for rural residents, men, informally employed and those without insurance. Infants were likely to have less utilisation that those above 60 years. Those in poorest economic quintile were likely to have greater utilisation than the better-off quintiles.

In terms of type of provider used, 67% [62.9%-70.8%] utilized public hospitals in 2020 and the rest used private hospitals. In 2019, the share of public hospitals in hospitalisations was 60.1% [56.8%-63.2%].

### Out of pocket expenditure on hospitalisation

Table 2 provides the mean OOPE per hospitalisation episode for different category of diseases for 2019 and 2020.

**Table 2:** Mean OOPE per hospitalisation (in INR, at 2019 prices) by type of disease, with 95% CI

**Disease Category**	**Mean OOPE per hospitalisation (in INR)**	
	**2019**	**2020**
Communicable Diseases	7836 [4854–10818]	8757 [4904–12609]
Non Communicable Diseases	24,533 [13718–35348]	30,143 [18106–42181]
Maternal Care	7292 [5316–9268]	12,679 [7123–18236]
Injuries	46,237 [16920–75553]	27,439 [2580–52298]
Others	17,823 [10504–25141]	49,289 [4908–93670]
**Overall**	**16,165 [12275—20256]**	**19,322 [14420–24225]**

Median OOPE in private sector doubled in 2020 from the 2019 level (Table 3).

**Table 3:** Mean and Median OOPE per hospitalisation (in INR, at 2019 prices) by type of provider, with 95% CI

**Type of Provider**	**Mean OOPE (INR)**		**Median OOPE (INR)**	
	**2019**	**2020**	**2019**	**2020**
**Public Hospitals**	4141 [3044- 5239]	4324 [1822- 6826]	500 [500–700]	500 [410–600]
**Private Hospitals**	34,989 [24978- 44999]	47,572 [35259–59884]	10,000 [10000–14000]	20,237 [16380–28665]
**Overall**	16,165 [12275—20256]	19,322 [14420–24225]	2000 [1500–2386]	1500 [1000–2091]

OLS regression for size of OOPE showed that the size of OOPE was likely to be larger for hospitalisations in private hospitals as compared to public facilities. Hospitalisations of men were likely to incur greater OOPE compared to women (Table 4). Those in richest quintile compared to the poorest, the graduates compared to the illiterate and the informally employed compared to the formally employed were likely to incur greater OOPE. Size of OOPE was not found to be associated with the year (2020 compared to 2019). Enrolment under national health insurance scheme, PMJAY was not associated with size of OOPE.

**Table 4:** OLS Regression for OOPE

Number of Observations:1381	**Coef**	R-Square		0.1496	
**Out of Pocket Expenditure**		**Std. Err**	***P* Value**	**95% Conf. Interval**	
**Place**					
Urban	1				
Rural	3471	4312	0.42	-4987	11,929
**Hospital Type**					
Private	1				
Public	-27,390	3536	0.00	-34,327	-20,452
**Age**					
< 1 years	1				
1–4 years	8340	24,505	0.73	-39,731	56,411
5–14 Years	3695	24,671	0.88	-44,703	52,094
15–48 Years	13,284	23,938	0.58	-33,677	60,244
49–59 Years	20,734	24,377	0.40	-27,087	68,556
> 60 Years	14,298	24,187	0.56	-33,149	61,746
**Sex**					
Male	1				
Female	-9781	3744	0.01	-17,125	-2437
**Caste**					
ST	1				
SC	8698	5413	0.11	-1920	19,316
OBC	18	3960	1.00	-7749	7786
Others	13,912	9912	0.16	-5532	33,356
**Occupation**					
Formal Sector	1				
Self-Employed	12,956	5513	0.02	2140	23,771
Informal Sector	13,538	5754	0.02	2250	24,825
Unemployed	41,205	20,922	0.05	162	82,248
Others	8836	19,039	0.64	-28,514	46,185
**Education**					
No Literate	1				
Primary	4082	4373	0.35	-4496	12,660
High school	6888	5788	0.23	-4465	18,242
Graduation and above	11,221	5590	0.05	255	22,187
**Household Expenditure Quintile**					
Q1 (Poorest)	1				
Q2 (Poor)	-726	4840	0.88	-10,220	8769
Q3 (Middle)	2443	5007	0.63	-7380	12,266
Q4 (Rich)	919	5373	0.86	-9621	11,459
Q5 (Richest)	13,287	5378	0.01	2737	23,837
**Disease**					
Communicable Disease	1				
NCD	10,564	4201	0.01	2323	18,806
Maternal	4059	4754	0.39	-5268	13,385
Emergency	16,397	6623	0.01	3403	29,390
Others	-494	7297	0.95	-14,809	13,821
**Year**					
2019	1				
2020	3439	3318	0.30	-3070	9947
**Duration of hospitalisation**	2083	316	0.00	1464	2703
**Insurance**					
Enrolled in PMJAY	1				
Not enrolled in PMJAY	4624	3514	0.19	-2270	11,518

### Catastrophic health expenditure (CHE) for hospitalisation

The findings on different measures used for CHE are given in Table 5. CHE in private hospitals was several times greater than public hospitals.

**Table 5:** CHE25, CHE10 and CHE40 for hospitalisation [in INR] by type of provider, with 95% CI

**Type of Provider**	**CHE25**		**CHE10**		**CHE40**	
	**2019**	**2020**	**2019**	**2020**	**2019**	**2020**
**Public Hospitals**	7.1 [5.2–9.5]	10.9 [7.6–15.3]	14.7 [12–17.9]	18.4 [14.2–23.6]	12.2 [9.7–15.4]	32.1 [26.7–37.9]
**Private Hospitals**	39.4 [34.5–44.6]	56.2 [48.8–63.4]	61.6 [56.5–66.5]	78.4 [71.7–83.9]	56.2 [50.9–61.4]	72.7 [65.6–78.8]
**Overall**	19.8 [17.3–22.5]	28.9 [24.9–33.8]	33.3 [30.3–36.4]	42.3 [37.8–47]	30.1 [27.1–33.1]	48.19 [43–52.8]

Probit regression for CHE40 showed that it was likely to be greater in year 2020 compared to 2019. Those utilizing private hospitals were also more likely to have CHE40 than public hospitals (Table 6). The poor were more likely to incur CHE40 than the rich. Longer duration hospitalisations were likely to cause greater CHE40. Hospitalisation for NCDs and injuries were associated with greater CHE40. Enrolment under PMJAY was not significantly associated with CHE40.

**Table 6:** Probit Regression for CHE40

Number of Observations:	1308	Pseudo R2:		0.2644	
**CHE40**	**Coef**	**Std. Err**	***P* Value**	**95% Conf. Interval**	
**Place**					
Urban	1				
Rural	-0.07	0.11	0.53	-0.29	0.15
**Hospital Type**					
Private	1				
Public	-1.39	0.09	0.00	-1.57	-1.20
**Age**					
< 1 years	1				
1–4 years	0.70	0.76	0.36	-0.80	2.19
5–14 Years	0.69	0.76	0.37	-0.81	2.19
15–48 Years	0.70	0.75	0.35	-0.77	2.17
49–59 Years	0.82	0.76	0.28	-0.66	2.30
> 60 Years	0.66	0.75	0.38	-0.82	2.13
**Sex**					
Male	1				
Female	0.06	0.10	0.51	-0.13	0.25
**Caste**					
ST	1				
SC	0.21	0.15	0.16	-0.08	0.50
OBC	-0.05	0.11	0.65	-0.26	0.16
Others	0.20	0.25	0.42	-0.29	0.69
**Occupation**					
Formal Sector	1				
Self-Employed	-0.09	0.14	0.51	-0.38	0.19
Informal Sector	-0.01	0.15	0.96	-0.30	0.29
Unemployed	1.12	0.56	0.04	0.03	2.21
Others	1.40	0.53	0.01	0.36	2.44
**Education**					
No Literate	1				
Primary	0.09	0.11	0.41	-0.13	0.32
High school	-0.02	0.15	0.89	-0.32	0.28
Graduation and above	0.03	0.15	0.83	-0.26	0.32
**Household Expenditure Quintile**					
Q1 (Poorest)	1				
Q2 (Poor)	-0.55	0.13	0.00	-0.80	-0.29
Q3 (Middle)	-0.42	0.13	0.00	-0.67	-0.16
Q4 (Rich)	-0.47	0.15	0.00	-0.75	-0.18
Q5 (Richest)	-0.91	0.15	0.00	-1.20	-0.61
**Disease**					
Communicable Disease	1				
NCD	0.24	0.11	0.03	0.02	0.45
Maternal	-0.03	0.13	0.85	-0.28	0.23
Emergency	0.50	0.17	0.00	0.17	0.83
Others	0.25	0.18	0.16	-0.10	0.60
**Year**					
2019	1				
2020	0.52	0.09	0.00	0.34	0.69
**Duration**	0.06	0.01	0.00	0.05	0.08
**Insurance**					
PMJAY	1				
No PMJAY	0.06	0.09	0.51	-0.12	0.24

### PSM model

PSM showed that OOPE was not affected by enrolment under PMJAY (Table 7). It also showed that CHE40 was not affected by enrolment under PMJAY (Table 7).

**Table 7:** Effect of enrolment under PMJAY on OOPE, CHE40 and CHE25 for Hospital Care – Results of Regression (OLS and Probit) and PSM

Outcome Variable	OLS Model		Probit Model		PSM Model [ATET]	
	Coeff	*P*-value	Coeff	*P*-value	Coeff	*P*-value
OOPE	-4624	0.19			-2147	0.52
CHE40			-0.06	0.51	0.03	0.30
CHE25			0.10	0.29	0.03	0.31

The above analyses were repeated for CHE25 but the pattern of results remained similar (Table 7).

The above analysis was repeated to find the effect of all PFHI schemes on OOPE and CHE, but the pattern of results remained similar.

## Discussion

While it is essential to understand the gaps in access to treatment for Covid-19 during the pandemic, it is of crucial importance that the changes experienced in meeting other healthcare needs also get assessed. The current study is the first such assessments in India that used a population based survey. This is also one of the first studies in India based on panel data of household surveys to measure healthcare utilisation and catastrophic health expenditure. This is also the first study to evaluate the role of PFHI in the context of non-Covid care during the pandemic.

The current study found that the utilisation of inpatient care declined significantly during the pandemic. Compared to 2019, the hospitalisations declined by 37% in 2020. The decline was wide-ranging across various healthcare needs and not limited to elective procedures. This confirms the concerns expressed about decrease in healthcare utilisation during the pandemic [2, 15, 16, 31]. What could be the potential reasons? One factor could be the restrictions on movement during lockdowns. Indian government had imposed very stringent lockdowns and curfew lasting several months [32, 33]. The availability of transport also suffered due to the pandemic related lockdowns [15]. There were hardly any arrangements made by the government for transport of non-Covid patients to hospitals. The existing fleet of government ambulances was also diverted towards transportation of Covid-19 infected cases. Another factor could be related to diversion of existing hospital capacity for Covid-19 care. While the government started a few new facilities to address hospitalisations needed of Covid-19 cases, it seems the enhancement of capacity was not sufficient [34]. Covid-19 related duties left the health workforce exhausted. The fear among healthcare workers of catching Covid-19 infection could have also impacted utilisation [31]. Researchers have reported that a large proportion among the private hospitals had stopped their services for some months during the pandemic [35]. The decline in hospitalisations in private sector in the current study indicates a similar possibility. Indian government had invoked the national law on essential services to ensure that the public hospitals stay operational during the pandemic but it was not imposed strictly for the private providers [34]. Another factor might be the fear among patients of catching Covid-19 infection while visiting hospitals [15]. Many patients postponed their treatment or underplayed their symptoms because of such fear. Globally, the above trends were pointed out early in the pandemic and governments were called upon to address the situation with appropriate policies [36]. The call was to create healthcare capacities to meet both kinds of needs – for Covid-19 treatment and non-Covid essential care [37]. There was specific guidance available for priority services to be maintained in resource constrained settings [38]. It seems that Indian government could not find adequate answers to the multiple challenges posed by the pandemic. Imposing stringent lockdowns remained the highlight of the government response and it made things worse for those needing urgent healthcare. A qualitative study from Nepal, a neighbouring country of India, has reported that unaffordability of care, closure of facilities and disruption in transport were key causes of reduced utilisation of healthcare during the pandemic [18].

The main findings of the current study were in terms of changes in OOPE and the incidence of catastrophic health expenditure for non-Covid hospitalisations during the pandemic. The current study found that incidence of catastrophic expenditure (CHE) on hospitalisations was significantly greater during the pandemic in 2020 as compared to 2019. Persons from poorer households were more likely to incur CHE than the economically better off. The increased chances of incurring catastrophic expenditure in 2020 could be due to the combined effect of decrease in income during the pandemic and the increase in OOPE. The overall mean OOPE increased by 19.5% from 2019 to 2020.

A small increase in OOPE was expected due to the additional cost incurred by private hospitals on personal protective equipment during the pandemic. However, the mean OOPE in private hospitals increased sharply, by 30% from 2019 to 2020. The increase in OOPE for private hospitals cannot be explained by the cost of personal protective equipment. Though the demand reduced, the private hospitals did not reduce their charges. The increase in OOPE in private sector could be related to price gouging, as reported by studies from India and other LMICs [39–41]. Price gouging refers to over-charging by hospitals from the patients at a time of crisis i.e. when the patients are more vulnerable. Utilisation in private hospitals was found to be the main determinant of size of OOPE and CHE. Mean OOPE per hospitalisation episode in private hospitals was eight times greater than in public hospitals in 2019 and the ratio worsened in 2020.

There was no new policy response from the government to protect the poor from CHE. The publicly funded health insurance remained the main policy for financial protection. However, the current study found that enrolment under PMJAY or other health insurance schemes was not effective in reducing chances of incurring CHE. The enrolment under PFHI schemes increased but it could not fulfil its promise of free care. The failure of PMJAY in controlling OOPE in private sector could be related to long-standing problem of double-billing under publicly funded health insurance in India [23, 42, 43]. Contracting was ineffective in ensuring adherence of private hospitals to agreed prices [23, 42].

Not much is known so far about the changes in OOPE for non-Covid healthcare during the pandemic in other LMICs. A large number of LMICs have implemented PFHI schemes [44]. A systematic review of PFHI in LMICs found that evidence of such schemes in meeting the goal of financial protection was inadequate [44]. A subsequent systematic review found many studies reporting positive impact of PFHI, though it also reported studies showing poor impact [45]. In the South Asian and South East Asian context, recent studies from India, Indonesia, Vietnam and Philippines have shown that increasing population coverage under PFHI has not been effective in protecting the people from high OOPE [46, 47]. The need of offering financial protection to the population is most evident in these nations but so is the limited success in achieving it. Limited capacity of the governments to handle contracting and the gaps in regulation of private providers come across as key challenges in such countries [42, 46].

The high incidence of CHE during the pandemic reflects the long standing structural problems in the Indian health system. Healthcare was source of financial risk and impoverishment for the poorer sections in the country before the pandemic. During the pandemic, the patients faced further asymmetry in power in relation to the providers. The policy makers failed to bring about a consensus strategy to provide affordable healthcare during the crisis. The existing policy of PFHI was not effective to start with and its performance worsened during the pandemic. A key feature of Indian PFHI is its reliance on for-profit private hospitals for delivery of services. This is a feature that seems to be common in countries with poorly effective PFHI like India and Indonesia [42, 47]. Further research is recommended to examine this aspect in depth in LMIC contexts.

India has a poorly regulated private healthcare sector. Private hospitals are non-transparent in their billing and there is no price regulation [39, 48, 49]. During the Covid-19 pandemic, the government tried to impose some price caps for treatment of the Covid-19 infected cases. The above attempt was not successful as the governments lacked the will to enforce the regulation. Private providers are very powerful in India and they exert huge influence on implementation of the government policies [50, 51]. Some have advocated that the governments should purchase care for Covid-19 from private sector [51–53]. In the context of the pandemic, there have been suggestions to promote the role of private sector further in Indian healthcare [54, 55]. The current study suggests that such strategies are unlikely to succeed in the Indian context.

Care in public hospitals was able to provide financial protection for most of the hospitalisations. It suggests that ways need to found to increase their share in healthcare utilisation. In addition, policy makers need to find ways to regulate the private hospitals. While restructuring the overall health system can be a long term goal, a strategy needs to be devised to promote affordable care during the crisis by bringing together the stakeholders from public and private sectors.

### Limitations

Quality of healthcare is an important dimension but it was beyond the scope of the study. Severity of illness is also important but it could not be covered in the study. The study did not collect data on morbidity or the number of people requiring hospitalisation. Therefore, the study could not compare the numbers of actual hospitalisations against the required hospitalisations. Data on the household expenditure including the food and non-food components was collected through a short set of questions. This could have resulted in respondents providing approximate figures for household expenditure but we believe that it did not affect the overall pattern of results.

## Conclusions

The access to hospital care for non-Covid needs suffered significantly during the pandemic. The fall in healthcare utilisation during the pandemic suggests that the government response of imposing heavy restrictions may be harmful during such situations. The Covid-19 pandemic also exposed the country’s fragile health system. Occurrence of catastrophic expenditure increased for hospital utilisation during the pandemic. Publicly funded insurance schemes like PMJAY though designed to cover hospitalisation costs, remained ineffective in controlling OOPE in private hospitals during the pandemic. Utilisation in private sector was a key determinant of catastrophic health expenditure.

There is a need to find ways to regulate the private hospitals in India and to limit overcharging. Implementation of strategies to increase the share of public sector in healthcare utilisation can help in reducing OOPE. The overall health system of the country needs to be strengthened to address the challenges posed by such emergencies and a consensus strategy needs to be evolved by involving both public and private sectors.

## Supplementary Information

**Additional file 1: Supplementary Information File S1.**List of Study Variables.

**Additional file 2: Supplementary Information File S2. **Socio-demographic Profile of the Sample.

**Additional file 3: Supplementary Information File S3.**Logistic Regression for Hospital Utilisation.

## Data Availability

The datasets used and/or analyzed during the current study are available from the corresponding author and State Health Resource Centre, Chhattisgarh on reasonable request.

## Abbreviations

CHE: Catastrophic Health Expenditure
INR: Indian Rupees
LMICs: Low and Medium Income Countries
OOPE: Out-of-pocket expenditure
PFHI: Publicly Funded Health Insurance
PMJAY: Pradhan Mantri Jan Arogaya Yojana

## References

[1] Paremoer L, Nandi S, Serag H, Baum F (2021). COVID-19 pandemic and the social determinants of health. BMJ.

[2] Singh A, Deedwania P, Vinay K, Chowdhury AR, Khanna P (2020). Is India's health care infrastructure sufficient for handling COVID 19 pandemic?. Int Arch Public Health Community Med.

[3] Hebbar PB, Sudha A, Dsouza V, Chilgod L, Amin A (2020). Healthcare delivery in India amid the Covid-19 pandemic: challenges and opportunities. Indian J Med Ethics.

[4] World Health Organization. Maintaining essential health services: operational guidance for the COVID-19 context: interim guidance, 1 June 2020. World Health Organization. Available from: https://apps.who.int/iris/handle/10665/332240.

[5] Moynihan R, Sanders S, Michaleff ZA (2021). Impact of COVID-19 pandemic on utilisation of healthcare services: a systematic review. BMJ Open.

[6] Santi L, Golinelli D, Tampieri A, Farina G, Greco M, Rosa S (2021). Non-COVID-19 patients in times of pandemic: emergency department visits, hospitalizations and cause-specific mortality in Northern Italy. PLoS One.

[7] De Rosa S, Spaccarotella C, Basso C, Calabrò MP, Curcio A, Filardi PP (2020). Reduction of hospitalisations for myocardial infarction in Italy in the COVID-19 era. Eur Heart J.

[8] Lee KD, Lee SB, Lim JK, Kang YM (2020). Providing essential clinical care for non-COVID-19 patients in a Seoul metropolitan acute care hospital amidst ongoing treatment of COVID-19 patients. J Hosp Infect.

[9] Kalanj K, Marshall R, Karol K, Tiljak MK, Oreskovic S (2021). The impact of COVID-19 on hospital admissions in Croatia. Front Public Health.

[10] Birkmeyer JD, Barnato A, Birkmeyer N, Bessler R, Skinner J (2020). The impact of the COVID-19 pandemic on hospital admissions in the United States. Health Aff.

[11] Abdela SG, Berhanu AB, Ferede LM, van Griensven J (2020). Essential healthcare services in the face of COVID-19 prevention: experiences from a referral hospital in Ethiopia. Am J Trop Med Hyg.

[12] Kumanan T, Rajasooriyar C, Guruparan M, Sreeharan N (2020). The Impact of COVID-19 on the delivery of critical health care: experience from a non–high-income country. Asia-Pacific J Public Heal.

[13] Adhikari KJN, Beane A, Devaprasad D, Fowler R, Haniffa R (2021). Impact of COVID-19 on non-COVID intensive care unit service utilization, case mix and outcomes: a registry-based analysis from India. Wellcome Open Res.

[14] Vaishya R, Sibal A, Kumar PS (2021). Severe impact of COVID-19 pandemic on non-COVID patient care and health delivery: an observational study from a large multispecialty hospital of India. Indian J Med Sci.

[15] Goyal M, Singh P, Singh K, Shekhar S, Agrawal N, Misra S (2021). The effect of the COVID-19 pandemic on maternal health due to delay in seeking health care: experience from a tertiary center. Int J Gynecol Obstet.

[16] Raman R, Rajalakshmi R, Surya J, Ramakrishnan R, Sivaprasad S, Conroy D (2021). Impact on health and provision of healthcare services during the COVID-19 lockdown in India: a multicentre cross-sectional study. BMJ Open.

[17] Ahmed SAKS, Ajisola M, Azeem K, Bakibinga P, Chen Y-F, Choudhury NN (2020). Impact of the societal response to COVID-19 on access to healthcare for non-COVID-19 health issues in slum communities of Bangladesh, Kenya, Nigeria and Pakistan: results of pre-COVID and COVID-19 lockdown stakeholder engagements. BMJ Glob Heal.

[18] Singh DR, Sunuwar DR, Shah SK, Karki K, Sah LK, Adhikari B (2021). Impact of COVID-19 on health services utilisation in Province-2 of Nepal: a qualitative study among community members and stakeholders. BMC Health Serv Res.

[19] World Health Organization. (2021). Second round of the national pulse survey on continuity of essential health services during the COVID-19 pandemic: January-March 2021: interim report, 22 April 2021. World Health Organization. https://apps.who.int/iris/handle/10665/340937.

[20] Government of India. About Pradhan Mantri Jan Arogaya Yojana (PM-JAY). [cited 11 April 2021] Available from: https://www.pmjay.gov.in/about-pmjay

[21] Garg S, Bebarta KK, Tripathi N (2020). Performance of India’s national publicly funded health insurance scheme, Pradhan Mantri Jan Arogaya Yojana (PMJAY), in improving access and financial protection for hospital care: Findings from household surveys in Chhattisgarh state. BMC Public Health.

[22] Key Indicators of Social Consumption in India: Health. NSS 75th round. July 2017-June 2018.Govenrment of India, Ministry of Statistics and Programme Implementation. National Statistics Office. http://mospi.nic.in/sites/default/files/publication_reports/KI_Health_75th_Final.pdf

[23] Garg S, Tripathi N, Ranjan A, Bebarta KK (2021). Comparing the average cost of outpatient care of public and for-profit private providers in India. BMC Health Serv Res.

[24] Government Of Chhattisgarh. Covid-19 Media Bulletin 31st December 2020. Available at: cghealth.nic.in. Accessed on 4th January, 2021.

[25] Johns Hopkins University of Medicine – Corona Virus Resource Centre. Daily confirmed new cases. Available at: https://coronavirus.jhu.edu/data/new-cases. Accessed 16 January 2022.

[26] Government of Chhattisgarh. Guidelines for Treatment of COVID-19 cases in Private Hospitals. Department of Health and Family Welfare. 2020. Available at: http://cghealth.nic.in/ehealth/COVID19/pages/index.html. Accessed 23rd March 2021.

[27] Government of Chhattisgarh. Guidelines for Home Isolation of COVID-19 cases. Department of Health and Family Welfare. 2020. Available at: http://cghealth.nic.in/ehealth/COVID19/pages/index.html. Accessed 23rd March 2021.

[28] Government of Chhattisgarh. Dr. Khoobchand Baghel Swasthya Sahayata Yojana (DKBSSY). cited 11 April 2021] Available from: https://dkbssy.cg.nic.in/

[29] Labour Bureau. Statistical Data for Labour – Index Numbers. Ministry of Labour, Government of India. [cited 11 April 2021] Available form: http://labourbureau.gov.in/LBO_indtab_Feb_2021.pdf

[30] Wagstaff A, Doorslaer E (2003). Catastrophe and impoverishment in paying for healthcare: with applications to Vietnam 1993–98. Health Economics.

[31] George CE, Inbaraj LR, Rajukutty S, De Witte LP (2020). Challenges, experience and coping of health professionals in delivering healthcare in an urban slum in India during the first 40 days of COVID-19 crisis: a mixed method study. BMJ Open.

[32] The Lancet (2020). India under COVID-19 lockdown. Lancet.

[33] Kandeger A, Guler HA, Egilmez U, Guler O (2018). Psychological impact of COVID-19 lockdown: an online survey from India. Indian J Psychiatry.

[34] Government of Chhattisgarh (2020). evelopment of Dedicated Hospitals for Covid-19. Department of Health and Family Welfare.

[35] David Williams O, Yung KC, Grépin KA (2021). The failure of private health services: COVID-19 induced crises in low- and middle-income country [LMIC] health systems. Glob Public Health.

[36] Rosenbaum L (2020). The untold toll — the pandemic’s effects on patients without Covid-19. N Engl J Med..

[37] Goulabchand R, Claret PG, Lattuca B (2020). What if the worst consequences of COVID-19 concerned non-COVID patients?. J Infect Public Health.

[38] Blanchet K, Alwan A, Antoine C, Cros MJ, Feroz F, AmsaluGuracha T (2020). Protecting essential health services in low-income and middle-income countries and humanitarian settings while responding to the COVID-19 pandemic. BMJ Glob Heal.

[39] Nandi S, Schneider H (2019). When state-funded health insurance schemes fail to provide financial protection: an in-depth exploration of the experiences of patients from urban slums of Chhattisgarh. India Glob Public Health.

[40] Thiagarajan K (2020). Covid-19 exposes the high cost of India’s reliance on private healthcare. BMJ.

[41] Hellowell M, Okafor Y. Understanding the impact of COVID-19 on the private health sector in Africa. Posted on March 9, 2021 by BMJ GH Blogs. [Cited 2021April 21]. Available from:

[42] Garg S, Chowdhury S, Sundararaman T (2019). Utilisation and financial protection for hospital care under publicly funded health insurance in three states in Southern India. BMC Health Serv Res.

[43] Reshmi B, Unnikrishnan B, Rajwar E (2021). Impact of public-funded health insurances in India on health care utilisation and financial risk protection: a systematic review. BMJ Open.

[44] Acharya A, Vellakkal S, Taylor F, Masset E, Satija A, Burke M, et al. The impact of health insurance schemes for the informal sector in low- and middle-income countries: a systematic review. World Bank Res Obs. Oxford University Press; 2013;28:236–266.

[45] Erlangga D, Suhrcke M, Bloor K, Ali S (2019). The impact of public health insurance on health care utilisation, financial protection and health status in low- and middle-income countries: a systematic review. PLoS One.

[46] Hanson K. Moving towards a purchasing model in low and middle income countries? RESYST Consortium. Fenruary 2016. Available at: http://resyst.lshtm.ac.uk @RESYSTresearch. Accessed 1 Nov 2021.

[47] Pratiwi AB, Setiyaningsih H, Kok MO (2021). Is Indonesia achieving universal health coverage? Secondary analysis of national data on insurance coverage, health spending and service availability. BMJ Open.

[48] Bhat R (1999). Characteristics of private medical practice in india: a provider perspective. Health Policy Plan.

[49] Sheikh K, Prasanna SS, Hort K (2015). What explains regulatory failure? analysing the architecture of health care regulation in two Indian States. Health Policy Plan.

[50] Mackintosh M, Channon A, Karan A, Selvaraj S, Zhao H, Cavagnero E (2016). What is the private sector? Understanding private provision in the health systems of low-income and middle-income. Lancet.

[51] Sanders D, Nandi S, Labonte R, Vance C, Damme WV (2019). From primary health care to universal health coverage—one step forward and two steps back. Lancet.

[52] Olalere N (2020). ause and reflect, July reflections, SPARC.

[53] Tsilaajav T, Mathauer I, de Oliveira Cruz V (2020). Purchasing health services to respond to COVID-10 How to involve and contract private sector providers in the South-East Asia Region?.

[54] Mathauer I, Dkhimi F, Townsend M (2020). Adjustments in health purchasing as part of the COVID-19 health response: results of a short survey and lessons for the future.

[55] NITI Ayog (2019). Health system for a new india: building blocks – potential pathways to reform.

[56] Sarwal R, Prasad U, Madangopal K, Kalal S, Kaur D, Kumar A, Regy P, Sharma J (2021). Investment Opportunities in India’s Healthcare Sector.

