# Supplementary material for: Household expenditure on non-Covid hospitalisation care during the Covid-19 pandemic and the role of financial protection policies in India
Source: Arch Public Health. 2022 Apr 2;80:108. doi: 10.1186/s13690-022-00857-8 (PMC8976164; doi:10.1186/s13690-022-00857-8)
Supplement: Supplementary file 1 — Additional file 1: Supplementary Information File S1.List of Study Variables. [file 13690_2022_857_MOESM1_ESM.docx]

**Supplementary Information File S1**

**List of Study Variables**

| **Variable Name** | **Variable Description** | **Category** |
| --- | --- | --- |
| Sex | Sex of Individual | Male |
|  |  | Female |
| Education Category |  | Not Literate |
|  |  | Primary |
|  |  | Higher Secondary |
|  |  | Graduate or above |
| Social Group | Social Group/Caste of Individual | Scheduled Tribes |
|  |  | Scheduled Castes |
|  |  | Other Backward Classes |
|  |  | Others |
| Per-Capita HH Expenditure Quintile | Quintiles of Per-capita Consumption Expenditure of Individual's Household | 1:Poorest |
|  |  | 2:Poor |
|  |  | 3: Middle |
|  |  | 4: Rich |
|  |  | 5: Richest |
| Occupation | Occupation of the Household | Formal sector Job |
|  |  | Self-employed |
|  |  | Informal Work |
|  |  | Unemployed |
|  |  | Others |
| Age | Age Category of Individual | <1 year |
|  |  | 1-4 Years |
|  |  | 5-14 Years |
|  |  | 15-48 Years |
|  |  | 49-59 Years |
|  |  | 60 Years and above |
| Marital Status | Marital Status of Individual | Never Married |
|  |  | Currently Married |
|  |  | Widow/Widower |
|  |  | Separated |
| Place | Place of Residence of Individual | Rural |
|  |  | Urban |
| Hospitalised | Whether the individual was hospitalised in last one year | Yes |
|  |  | No |
| PFHI | Whether individual enrolled under any PFHI scheme | Yes |
|  |  | No |
| Insured under PMJAY | Whether individual enrolled under PMJAY scheme | PMJAY |
|  |  | No PMJAY |
| Type of Hospital | Type of Hospital in which hospitalisation took place | Public Hospital |
|  |  | Private Hospital |
| Disease Category | Category of Disease/Condition for which hospitalisation episode took place | Communicable Diseases |
|  |  | Non Communicable Diseases |
|  |  | Maternal |
|  |  | Emergency & Injury |
|  |  | Others |
| Duration | Duration of Hospitalisation Episode | |
| OOPE | Out of Pocket Expenditure (OOPE) at 2019 prices in INR | |
| CHE10 | Catastrophic Health Expenditure at 10% threshold (OOPE > 10% of Annual Consumption Expenditure of concerned Household) | Yes |
|  |  | No |
| CHE25 | Catastrophic Health Expenditure at 25% threshold (OOPE > 25% of Annual Consumption Expenditure of concerned Household) | Yes |
|  |  | No |
| NonFood Expenditure CHE40 | Catastrophic Health Expenditure at 40% threshold (OOPE > 40% of Annual Nonfood Consumption Expenditure of concerned Household) | Yes |
|  |  | No |
| Year | Year of Survey | 2019 |
|  |  | 2020 |
