# Supplementary material for: Household expenditure on non-Covid hospitalisation care during the Covid-19 pandemic and the role of financial protection policies in India
Source: Arch Public Health. 2022 Apr 2;80:108. doi: 10.1186/s13690-022-00857-8 (PMC8976164; doi:10.1186/s13690-022-00857-8)
Supplement: Supplementary file 2 — Additional file 2: Supplementary Information File S2. Socio-demographic Profile of the Sample. [file 13690_2022_857_MOESM2_ESM.docx]

**Supplementary Information File S2**

**Socio-demographic Profile of the Sample**

| **Characteristic** | | **2019** | **2020** |
| --- | --- | --- | --- |
|  |  | **(N=15470)** | **(N=14,926)** |
| Place of residence (Urban/Rural) | Urban | 21.4% | 21.1% |
|  | Rural | 78.6% | 78.9% |
| Household size | Mean with 95% CI | 5.9 | 5.7 |
| Occupation | Formal Sector | 10.6% | 7.1% |
|  | Self-Employed | 50.5% | 58.3% |
|  | Informal Sector | 37.8% | 34% |
|  | Unemployed | 0.4% | 0.2% |
|  | Others | 0.7% | 0.3% |
| Social Group (Caste) | ST | 33.6% | 35.3% |
|  | SC | 11.8% | 11.3% |
|  | OBC | 51.2% | 49.1% |
|  | Others | 3.4% | 4.4% |
| Sex of Individual | Male | 49.7% | 49.7% |
|  | Female | 50.3% | 50.3% |
| Age Category of Individual | < 1 years | 0.8% | 1.0% |
|  | 1-4 years | 7.7% | 6.6% |
|  | 5-14 Years | 18.2% | 18.2% |
|  | 15-48 Years | 56.7% | 57.7% |
|  | 49-59 Years | 8% | 8.1% |
|  | > 60 Years | 8.2% | 8.4% |
| Education of Individual | No Literate | 30.1% | 28.3% |
|  | Primary | 43.7% | 44.4% |
|  | High school | 11.6% | 11.9% |
|  | Graduation and above | 14.4% | 15.4% |
| Individuals Covered with Insurance | PMJAY | 45.5% | 52.4% |
|  | State scheme (MSBY) | 21.9% | 42.7% |
|  | Other | 0.7% | 4.7% |
|  | No Insurance | 32% | 0.25% |
| Per capita Annual Household expenditure (INR) | Mean | 14091 | 13190 |
|  | Median | 12000 | 10800 |
| Per capita Annual Non food expenditure (INR) | Mean | 5575 | 5380 |
|  | Median | 4000 | 3771 |
