# Supplementary material for: Household expenditure on non-Covid hospitalisation care during the Covid-19 pandemic and the role of financial protection policies in India
Source: Arch Public Health. 2022 Apr 2;80:108. doi: 10.1186/s13690-022-00857-8 (PMC8976164; doi:10.1186/s13690-022-00857-8)
Supplement: Supplementary file 3 — Additional file 3: Supplementary Information File S3.Logistic Regression for Hospital Utilisation. [file 13690_2022_857_MOESM3_ESM.docx]

**Supplementary Information File S3**

**Logistic Regression for Hospital Utilisation**

| Number of Observations : | 30006 |  |  |  |  |
| --- | --- | --- | --- | --- | --- |
|  |  |  |  |  |  |
| **Hospitalisation** | **Odds Ratio** | **Std. Err.** | **P Value** | **95% Conf. Interval** | |
| **Place** |  | | | | |
| Urban | 1 |  | | | |
| Rural | 1.44 | 0.12 | 0.00 | 1.22 | 1.71 |
| **Sex** |  | | | | |
| Male | 1 |  | | | |
| Female | 1.93 | 0.11 | 0.00 | 1.72 | 2.16 |
| **Age** |  | | | | |
| < 1 years | 1 |  | | | |
| 1-4 years | 1.32 | 0.53 | 0.48 | 0.60 | 2.91 |
| 5-14 Years | 0.59 | 0.24 | 0.20 | 0.27 | 1.31 |
| 15-48 Years | 1.93 | 0.75 | 0.09 | 0.90 | 4.15 |
| 49-59 Years | 1.63 | 0.65 | 0.22 | 0.75 | 3.55 |
| > 60 Years | 2.65 | 1.04 | 0.01 | 1.23 | 5.73 |
| **Caste** |  | | | | |
| ST | 1 |  | | | |
| SC | 1.18 | 0.11 | 0.07 | 0.99 | 1.41 |
| OBC | 0.94 | 0.06 | 0.34 | 0.83 | 1.07 |
| Others | 0.73 | 0.12 | 0.06 | 0.53 | 1.02 |
| **Occupation** |  | | | | |
| Formal Sector | 1 |  | | | |
| Self-Employed | 1.03 | 0.10 | 0.75 | 0.85 | 1.25 |
| Informal Sector | 0.80 | 0.08 | 0.02 | 0.65 | 0.97 |
| Unemployed | 1.57 | 0.57 | 0.22 | 0.77 | 3.21 |
| Others | 0.90 | 0.32 | 0.76 | 0.44 | 1.82 |
| **Education** |  | | | | |
| No Literate | 1 |  | | | |
| Primary | 1.14 | 0.09 | 0.12 | 0.97 | 1.33 |
| High school | 1.14 | 0.10 | 0.15 | 0.95 | 1.35 |
| Graduation and above | 1.19 | 0.12 | 0.08 | 0.98 | 1.44 |
| **Household Expendeture Quintile** |  | | | | |
| Q1 (Poorest) | 1 |  | | | |
| Q2 (Poor) | 0.81 | 0.07 | 0.01 | 0.68 | 0.95 |
| Q3 (Middle) | 0.86 | 0.07 | 0.09 | 0.73 | 1.02 |
| Q4 (Rich) | 0.77 | 0.07 | 0.00 | 0.65 | 0.92 |
| Q5 (Richest) | 0.88 | 0.08 | 0.14 | 0.73 | 1.05 |
| **Insurance** |  | | | | |
| PMJAY | 1 |  | | | |
| No PMJAY | 0.97 | 0.06 | 0.67 | 0.86 | 1.10 |
| **YEAR** |  | | | | |
| 2019 | 1 |  | | | |
| 2020 | 0.45 | 0.04 | 0.00 | 0.38 | 0.54 |
